# Supplementary material for: Harnessing YouTube in advancing biodiversity conservation efforts and awareness across Africa
Source: PeerJ. 2025 Jun 12;13:e19545. doi: 10.7717/peerj.19545 (PMC12169165; doi:10.7717/peerj.19545)
Supplement: Supplemental Information 2 [file peerj-13-19545-s002.docx]

**Results of the Shapiro test**

According to the Shapiro test, the data was not normally distributed for views per day (vpd) (W = 0.19, p< 0.001), comments per day (cpd) (W = 0.12108, p-value < 0.001), and likes per day (lpd) (W = 0.076078, p-value < 0.001).

**Table S1** The Post hoc Dunn test indicates the significant differences in views per day (vpd) and likes per day (lpd) between countries (**(χ^2^),** and the Z-score indicates the statistical difference between countries with particular p-values.

| type | comparisons | chi2 | Z | P | P.adjusted |
| --- | --- | --- | --- | --- | --- |
| vpd | DRC - Egypt | 32.784 | 2.784 | 0.003 | 0.003 |
|  | DRC - Guinea Bissau | 32.784 | 2.327 | 0.010 | 0.010 |
|  | Egypt - Madagascar | 32.784 | -2.288 | 0.011 | 0.011 |
|  | Egypt - Nigeria | 32.784 | -2.638 | 0.004 | 0.004 |
|  | Guinea Bissau - Nigeria | 32.784 | -2.175 | 0.015 | 0.015 |
|  | DRC - others | 32.784 | 2.270 | 0.012 | 0.012 |
|  | Nigeria - others | 32.784 | 2.081 | 0.019 | 0.019 |
|  | DRC - Rwanda | 32.784 | 2.350 | 0.009 | 0.009 |
|  | Nigeria - Rwanda | 32.784 | 2.254 | 0.012 | 0.012 |
|  | DRC - South Africa | 32.784 | 2.174 | 0.015 | 0.015 |
|  | Nigeria - South Africa | 32.784 | 1.971 | 0.024 | 0.024 |
|  | DRC - Uganda | 32.784 | 3.938 | 0.000 | 0.000 |
|  | Kenya - Uganda | 32.784 | 3.253 | 0.001 | 0.001 |
|  | Madagascar - Uganda | 32.784 | 3.941 | 0.000 | 0.000 |
|  | Nigeria - Uganda | 32.784 | 3.579 | 0.000 | 0.000 |
|  | others - Uganda | 32.784 | 2.415 | 0.008 | 0.008 |
|  | South Africa - Uganda | 32.784 | 3.097 | 0.001 | 0.001 |
| lpd | DRC - Egypt | 27.598 | 2.792 | 0.003 | 0.003 |
|  | DRC - Guinea Bissau | 27.598 | 2.224 | 0.013 | 0.013 |
|  | DRC - Namibia | 27.598 | 2.113 | 0.017 | 0.017 |
|  | Egypt - Nigeria | 27.598 | -2.485 | 0.006 | 0.006 |
|  | DRC - others | 27.598 | 2.276 | 0.011 | 0.011 |
|  | DRC - Rwanda | 27.598 | 2.567 | 0.005 | 0.005 |
|  | Nigeria - Rwanda | 27.598 | 2.306 | 0.011 | 0.011 |
|  | DRC - South Africa | 27.598 | 2.362 | 0.009 | 0.009 |
|  | DRC - Uganda | 27.598 | 3.691 | 0.000 | 0.000 |
|  | Kenya - Uganda | 27.598 | 2.732 | 0.003 | 0.003 |
|  | Madagascar - Uganda | 27.598 | 2.954 | 0.002 | 0.002 |
|  | Nigeria - Uganda | 27.598 | 3.166 | 0.001 | 0.001 |
|  | others - Uganda | 27.598 | 2.065 | 0.019 | 0.019 |
|  | South Africa - Uganda | 27.598 | 2.444 | 0.007 | 0.007 |
|  | Uganda - Zimbabwe | 27.598 | -2.369 | 0.009 | 0.009 |

**Table S2**: The Post hoc Dunn test indicates the significant differences in views per day between high organism groups (**(χ^2^),** and the Z-score indicates the statistical difference between countries with particular p-values.

| **type** | **comparisons** | **chi2** | **Z** | **P** | **P.adjusted** |
| --- | --- | --- | --- | --- | --- |
| vpd | Amphibians - Fishes | 17.934 | -2.808 | 0.002 | 0.002 |
|  | Fishes - Insect | 17.934 | 2.260 | 0.012 | 0.012 |
|  | Amphibians - Mammals | 17.934 | -2.399 | 0.008 | 0.008 |
|  | Fishes - Plants | 17.934 | 2.035 | 0.021 | 0.021 |
|  | Fishes - Reptiles | 17.934 | 2.610 | 0.005 | 0.005 |
|  | Mammals - Reptiles | 17.934 | 2.210 | 0.014 | 0.014 |
| lpd | Amphibians - Birds | 15.592 | -2.156 | 0.016 | 0.016 |
|  | Amphibians - Fishes | 15.592 | -2.551 | 0.005 | 0.005 |
|  | Fishes - Plants | 15.592 | 1.983 | 0.024 | 0.024 |
|  | Birds - Reptiles | 15.592 | 2.276 | 0.011 | 0.011 |
|  | Fishes - Reptiles | 15.592 | 2.603 | 0.005 | 0.005 |
|  | Mammals - Reptiles | 15.592 | 2.057 | 0.020 | 0.020 |
| cpd | Amphibians - Fish | 14.274 | -2.261 | 0.012 | 0.012 |
|  | Fishes - Insect | 14.274 | 2.121 | 0.017 | 0.017 |
|  | Birds - Reptiles | 14.274 | 2.018 | 0.022 | 0.022 |
|  | Fishes - Reptiles | 14.274 | 2.534 | 0.006 | 0.006 |
|  | Mammals - Reptiles | 14.274 | 2.216 | 0.013 | 0.013 |
